# MURM-A*: An Improved A* Within Comprehensive Path-Planning Scheme for Cellular-Connected Multi-UAVs Based on Radio Map and Complex Network

**DOI:** 10.3390/s26030965

**Published:** 2026-02-02

**Authors:** Yanming Chai, Qibin He, Yapeng Wang, Xu Yang, Sio-Kei Im

**Affiliations:** 1 Faculty of Applied Sciences, Macao Polytechnic University, Macao SAR 999078, China; yanming.chai@mpu.edu.mo (Y.C.); p2316021@mpu.edu.mo (Q.H.); xuyang@mpu.edu.mo (X.Y.); 2 Macao Polytechnic University, Macao SAR 999078, China; marcusim@mpu.edu.mo

**Keywords:** unmanned aerial vehicle (UAV), multi-UAV, path planning, radio map, complex network, path-planning model, A-star

## Abstract

For the purpose of fulfilling the dual requirements of persistent cellular network connectivity and flight safety for cellular-connected Unmanned Aerial Vehicles (UAVs) operating in dense urban airspace, this paper presents an A*-oriented comprehensive path-planning scheme for multiple connected UAVs that integrates a radio map and complex network. Existing research often lacks rigorous processing of environmental map data, while the traditional A* algorithm struggles to simultaneously handle constraints such as obstacle avoidance, flight maneuverability, and multi-UAV path conflicts. To overcome these limitations, this study first constructs a path-planning model based on complex-network theory using environmental data and the radio map, clarifying the separation of responsibilities between environment representation and algorithmic search. On this basis, we proposed an improved A* algorithm for multi-UAV scenarios termed MURM-A*. Simulation results demonstrate that the proposed algorithm effectively avoids collisions with obstacles, adheres to UAV flight dynamics, and prevents spatial conflicts between multi-UAV paths, while achieving a joint optimization between path efficiency and radio quality. In terms of performance comparison, the proposed algorithm shows a marginal difference but ensures operational validity compared to traditional A*, exhibits a slightly increase in flight time but achieves a substantial reduction in radio-outage time compared to the Deep Reinforcement Learning (DRL) method. Furthermore, employing the path-planning model enables the algorithm to more accurately identify environmental information compared to directly using raw environmental maps. The modeling time is also notably shorter than the training time required for DRL methods. This study provides a well-structured and extensible systematic framework for reliable path planning of multiple cellular-connected UAVs in complex radio environments.

## 1. Introduction

**Figure 1:**
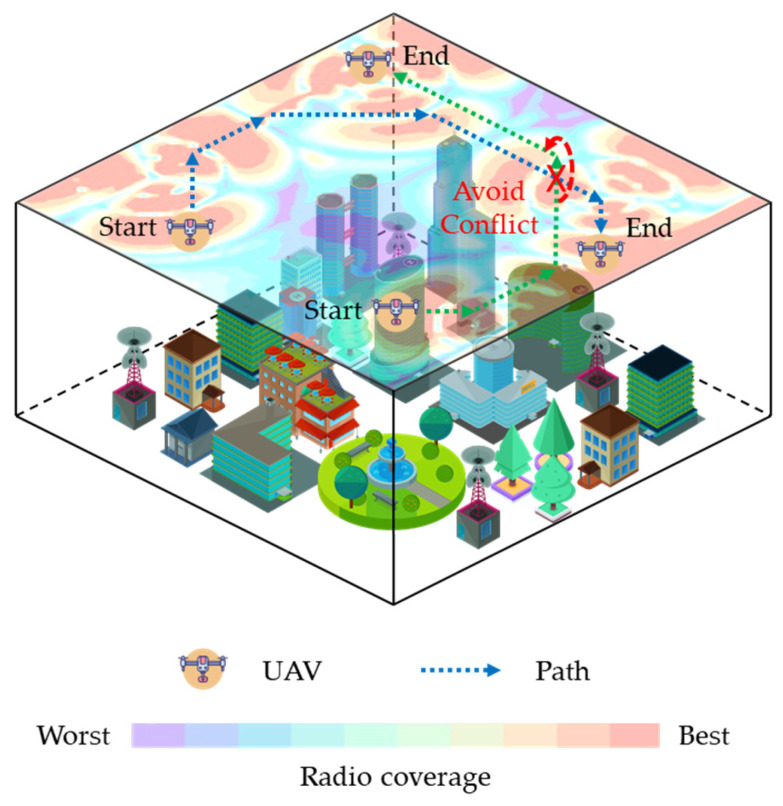
Visual representation of the research scenario for path planning of cellular-connected UAV in a radio map.

In recent years, cellular-connected UAVs [1] have been increasingly applied in tasks such as area inspection, logistics delivery, and emergency communication. These missions require UAVs not only to navigate safely from the start to the destination but also to maintain reliable cellular network connectivity throughout the flight in complex urban airspace while ensuring conflict-free paths among multiple UAVs. Radio maps graphically represent communication blind spots caused by building obstructions, thereby providing essential environmental information for communication-aware and safety-critical path planning. Figure 1 illustrates a research scenario for such planning, where a radio map is utilized to enable effective obstacle avoidance, sustained communication quality, and collision prevention under complex radio propagation conditions.

Although there is a substantial body of existing research on UAV path planning, significant shortcomings remain when addressing the aforementioned challenges. Firstly, the optimization objectives from most approaches often pursue only the shortest path while failing to simultaneously incorporate critical real-world constraints such as radio condition, flight maneuverability, and multi-UAV path conflicts.

Subsequently, in terms of methodology, complex networks, as a graph theory-based network structure, enable weighted path planning through graph search, making them one of the mainstream approaches [2]. Yet, research in this direction generally concentrates solely on proposing path-planning algorithms while neglecting the design of the path-planning model. A common practice involves deploying algorithms directly onto an environmental map or altering map data, creating a technical bottleneck reliant on a single map that serendipitously contains all necessary information. Such tight coupling between algorithm and environment map introduces several potential technical limitations: (1) Vulnerability to map data corruption; (2) Impaired pathfinding decisions when multiple maps are present, due to difficulties in accurately identifying or fusing heterogeneous map information; (3) A lack of generalization capability, necessitating complete remodeling and redesign whenever the map changes due to semantic updates, environmental changes, or scenario switches.

Furthermore, among path-planning algorithms in the field of complex networks, the A* algorithm, as a widely recognized and efficient heuristic search algorithm, has attracted extensive attention due to its simple principles, ease of implementation, and guarantee of finding an optimal path. Then again, paths generated by the traditional A* algorithm often exhibit abrupt turns that do not conform to UAV maneuverability, and the algorithm inherently lacks capabilities such as obstacle avoidance and multi-UAV conflict prevention. These limitations make it difficult to directly apply in high-reliability scenarios involving multiple connected UAVs.

Therefore, to address the issues present in existing research, it is necessary to design a path-planning model that is capable of integrating information from various maps, comprehensively reflecting factors such as communication quality and obstacle locations, to separate the algorithm from environmental mapping. Concurrently, the A* algorithm necessitates improvement so that its pathfinding incorporates UAV flight characteristics, obstacle avoidance capabilities, and multi-UAV conflict prevention. This allows for the seamless integration of real-world constraints while preserving the efficient search core of the A* algorithm.

This paper, focusing on the dense urban 3D airspace radio map as the research scenario, presents a conceptual comprehensive path-planning scheme for multiple UAVs based on an improved A* algorithm. The main contributions include:

1. Construction of an integrated path-planning model: Based on the environmental map and radio map from the specific scenario, we construct an innovative path-planning model using a complex network. This model provides algorithms with a clean, environment map-agnostic representation, featuring extensibility and short construction time. In simulations, the model construction time was substantially reduced compared to a DRL approach (approximately 2 s vs. 60 h).
2. Proposal of MURM-A* algorithm: Building on the path-planning model and traditional A*, we propose MURM-A*. By incorporating directional, obstacle, and multi-UAV conflict constraints into an optimized node expansion strategy, the algorithm plans optimal paths that strictly adhere to all specified constraints. Simulation results demonstrate the achievement of 0 obstacle collisions, 0 excessive turning angles, and 0 multi-UAV conflicts.
3. Comprehensive Simulation Verification: We designed comparative simulation experiments within a generated 3D dense urban model along with a corresponding radio map. The results indicate that compared to the DRL approach, the proposed scheme marginally increased the flight time (around 6%) while significantly reducing the outage duration (around 43%). The results validate the effectiveness and superiority of the proposed algorithm within the path-planning model under multiple constraints.

This study provides a structured, easily implementable, and extensible conceptual solution for multi-UAV path planning in complex radio environments, contributing positively to advancing the practical application of cellular-connected UAVs in dense urban areas.

## 2. Related Work

### 2.1. Cellular-Connected UAV Path Planning

In the field of path planning for cellular-connected UAVs, a substantial body of research has emerged. Existing algorithms can be primarily classified under two principal frameworks: complex networks and machine learning. The latter one is currently the mainstream direction. In particular, DRL methods, which learn through trial-and-error via continuous interaction between the agent and the environment to autonomously master optimal decision-making policies, have demonstrated exceptional environmental adaptability and potential for generalization to unknown scenarios.

Recent DRL research has largely focused on utilizing classic frameworks like Deep Q-Networks (DQN) and Deep Deterministic Policy Gradient (DDPG) [3], training reward mechanisms based on Markov Decision Processes to achieve real-time trajectory optimization [4]. This has led to numerous DQN-based improved algorithms for UAVs. Scholars such as Betalo et al. proposed a Multi-Agent Deep Q-Network algorithm (MADQN) to synchronously optimize dynamic charging and energy allocation strategies for laser-powered UAV path planning [5], later improving it to an MA-DDQN algorithm based on Dual Deep Q-Networks, further enhancing energy efficiency and task speed [6]. Chen et al. proposed a method based on an improved DDQN, enhancing planning performance through a composite action selection strategy and a distributed value network [7]. Kong et al. even integrated Artificial Potential Fields (APF) as prior knowledge into DQN, combining it with an adaptive exploration strategy and B-spline optimization, significantly improving the convergence speed and path smoothness [8]. However, these studies do not yet incorporate elements related to cellular connectivity.

Early work in this connectivity-aware direction started with simplified Line-of-Sight (LoS) channel modeling. Bulut et al. were among the first to study the trajectory optimization problem for UAVs experiencing unstable connections to ground cellular networks, requiring interruption duration to remain below a specific threshold, and proposed a linear programming solution with high computational complexity [9]. Challita et al. further optimized UAV flight trajectories in interference environments of coexisting aerial and terrestrial networks [10]. Gesbert et al. achieved UAV trajectory optimization in 3D scenarios considering network connectivity constraints [11]. Lee et al. proposed a DQN-based trajectory planning method for UAV base station networks, realizing 3D trajectory optimization and seamless transition from static to dynamic scenarios in UAV networking [12]. Then again, these studies still have not yet utilized radio maps.

Research on path planning for cellular-connected UAVs based on a radio map was first initiated by the Zeng team, who combined it with DRL to propose an integrated optimization tactic of radio connection and path navigation [13]. Hao et al. utilized a deep image prior framework to reconstruct a radio map, which enabled their enhanced DRL algorithm to substantially decrease the energy consumption [14]. Liu et al. employed an improved D3QN algorithm with a prioritized experience replay mechanism, combined with radio map construction, effectively balancing flight time and communication interruption time [15]. Zhou et al. combined interference fluid dynamics systems with radio maps and proposed a TD3 algorithm [16]. It should be noted that while machine learning methods offer flexible and rapid path-planning capabilities, they still face challenges in model training, such as insufficient generalization ability of trained models, high demands on computational resources, and lengthy, unstable training processes.

Within the research scope of complex networks, the path-planning problem is typically modeled as a complex system in graph theory. The research history began with classic graph search algorithms such as Dijkstra, Floyd, A*, Ant Colony Optimization (ACO), and Rapidly exploring Random Trees (RRT) [17,18]. However, such algorithms struggle to completely accommodate the actual flight constraints of UAVs in their traditional form; subsequent research has largely concentrated on their improvement and optimization. In discrete space path planning, the A* algorithm is mostly preferred for its efficiency. Ju et al. improved it to possess dynamic obstacle avoidance capabilities while guaranteeing solution optimality in specific scenarios [19]. Chen et al. significantly reduced redundant nodes of the traditional A* algorithm in 3D path planning and improved search efficiency by optimizing the heuristic function, introducing bidirectional search, and path tie-breaking strategies [20]. Other studies have improved the traditional A* algorithm from multiple angles by combining it with other domains. Zhang et al., integrating a feature attention mechanism, proposed the RFA* algorithm, which demonstrates more stable and efficient performance in complex environments [21]. Bai et al. fused A* with the Dynamic Window Approach (DWA), significantly enhancing path smoothness, safety, and real-time obstacle avoidance capability by preprocessing irregular obstacles and introducing an adaptive evaluation function [22]. Liu et al. proposed a model combining Digital Elevation Model (DEM) data with an improved A* algorithm, incorporating complex environmental factors like integrated terrain and weather threats into the heuristic search strategy [23]. In path planning within multidimensional and continuous state spaces, the research focus has mainly been on RRT and ACO. Gu et al. reduced the blindness of random sampling in RRT by optimizing the sampling strategy and introduced collision detection for effective obstacle avoidance [24]. Zhao et al. combined RRT* with the Artificial Potential Field (APF), introducing a local spherical space sampling strategy, which significantly improved convergence speed [25]. Guo et al. proposed the FC-RRT* algorithm, which controls sampling through a flight cost function, reducing encountered threats while achieving near-optimal path planning [26]. Zheng et al. deeply integrated motion planning networks with the RRT* algorithm, proposing a novel path-planning framework named MPN-RRT*. Through a 3D-to-2D dimensionality reduction strategy and a bidirectional neural planning mechanism, it significantly enhanced the real-time performance, optimized path length, and smoothness of UAV path planning in complex urban environments [27]. Konatowski et al. employed the ACO algorithm to optimize threat and flight costs [28]. Guan et al. used a hybrid of dual ant colonies and a genetic algorithm, significantly improving the convergence speed of path planning [29]. However, the aforementioned studies did not fully consider the strong dependence of cellular-connected UAVs on sustained and stable communication.

Addressing this limitation, Zhang et al., based on graph theory methods, transformed the UAV path-planning problem under connectivity quality constraints into a shortest path problem on a graph, aiming to minimize mission completion time [30]. Within the same context, Yang developed a method that iteratively applies geometric inequalities, significantly reducing computational complexity while enhancing path performance [31]. In an innovative integration, Chen et al. designed a graph-based path optimization framework constrained by radio conditions, utilizing radio maps as a core component of their methodology [32]. In general, the complex-network approach, built upon a mature graph-theoretic framework, has improved its practicality through various enhancement strategies and has gradually incorporated communication constraints in later stages of development. Yet, its primary focus remains on single-UAV path planning.

Beyond the two main categories discussed, research on other methodologies also exists. For instance, Carrese et al. employed a mixed-integer programming model to optimize paths and flight schedules for a scenario where UAVs monitor vehicle status [33]. Chu et al. discussed paradigms for applying particle swarm optimization metaheuristics to UAV-related problems [34].

### 2.2. Multi-UAV Path Planning

The aforementioned studies have predominantly focused on path planning for a single UAV, without addressing the design for multi-UAV systems. When the problem extends to multi-cellular-connected UAV systems, the core challenge shifts from finding an individual optimal path to resolving path conflicts and resource competition within a dynamic, cooperative environment. This paradigm shift renders many excellent single-UAV algorithms difficult to apply directly.

To address these challenges, research has similarly followed the two main technical paths of complex networks and machine learning. Methods based on complex networks typically employ either centralized or decoupled strategies. Centralized strategies model the entire fleet as a global optimization problem. While theoretically capable of achieving a system-wide optimal solution, their computational complexity grows exponentially with the number of UAVs, presenting a significant scalability bottleneck [35]. Decoupled strategies reduce the computational burden through a hierarchical approach and have become a common method. Hierarchical approaches themselves vary. Guo et al. proposed a hierarchical 4D dynamic planning strategy that first uses RRT* for cooperative planning, then employs APF for local collision avoidance against dynamic threats, effectively solving the spatiotemporal coordination and real-time obstacle avoidance problem for multiple UAVs in dynamic threat environments [36]. Lin et al. integrated the elastic band with an improved A* algorithm to form a hierarchical strategy that first performs global obstacle avoidance, then path smoothing, enhancing path smoothness and real-time planning capability for multiple UAVs in complex environments [37]. Du et al. adopted a decoupled approach where task areas are first assigned to each UAV, followed by independent path planning for each using the A*, significantly reducing computation time and memory usage in 3D search and rescue environments [38]. It is important to note, however, that such strategies often decouple path planning from coordination, typically resulting in suboptimal rather than optimal solutions.

Within machine learning methods, DRL has become a research hotspot due to its capability for autonomous learning and decision-making in complex environments. The APF-TD3 algorithm proposed by Wu et al. effectively mitigates the sparse reward problem in traditional reinforcement learning, significantly improving training efficiency and path quality in dynamic multi-obstacle environments [39]. Westheider et al. addressed the multi-UAV informative path-planning problem with a DRL method based on the COMA framework, demonstrating good generalization ability under varying team sizes and communication constraints [40]. Chen et al. focused on coverage path planning, proposing a multi-UAV cooperative algorithm based on Double Q-learning, which can complete full-coverage tasks across various terrains with lower total cost and smoother paths [41]. These studies collectively illustrate the flexibility and adaptability of machine learning in multi-UAV path planning, providing effective solutions for cooperative decision-making in complex scenarios.

Furthermore, within multi-UAV scenarios, a special case exists: Cooperative formation path planning. Unlike conventional multi-UAV scenarios with independent tasks, this case requires the UAV swarm to operate as a coordinated collective (e.g., in a specific formation), where path planning is deeply coupled with formation keeping, imposing extremely high demands on spatiotemporal consistency. This has led to its establishment as a distinct research direction. Graph-based methods often address this by introducing virtual structures or leader-follower models, transforming the formation control problem into a series of path-following problems [42], and employing algorithm frameworks capable of rapid replanning to coordinate multi-UAV paths [43]. However, the flexibility of such methods in dynamic environments still requires improvement. In DRL, Multi-Agent Reinforcement Learning frameworks are typically employed to achieve distributed cooperative decision-making through shared observations or independent policies. Xu et al. proposed a Feature Fusion Proximal Policy Optimization (FF-PPO) algorithm, which utilizes visual perception and target detection information to achieve multi-UAV cooperative planning without precise target locations in GPS-denied and communication-denied environments [44]. Luo et al. adopted a DQN architecture, jointly modeling computation offloading and flight trajectories as a Markov Decision Process to minimize uncertainty in the search area [45]. A comprehensive review indicates that existing methods in collaborative path planning have yet to achieve a satisfactory balance between control precision and algorithmic flexibility. Furthermore, balancing formation accuracy with algorithm convergence in large-scale swarms remains a significant challenge for this direction.

### 2.3. Common Issues

Taking an overarching view of the aforementioned research, some common issues can also be identified. Firstly, regarding application scenarios, most studies rely on overly simplified settings, which is evident in:

- Narrow Optimization Objectives: The existing path-planning schemes often consider only the single factor of minimizing the path length from start to end. Consequently, performance comparison experiments focus predominantly on the path length metric, neglecting to integrate critical constraints such as network coverage, obstacle avoidance, and UAV flight dynamics.
- Unrealistic Simulation Environments: The simulated environments are frequently simplistic, featuring building layouts that are either overly regular or excessively sparse, resulting in a significant discrepancy from real-world conditions.

Furthermore, for complex-network-based methods, while their advantages lie in short modeling time and strong adaptability to environmental changes, related research often suffers from inadequate experimental environment design, obscuring these strengths. This manifests specifically in:

- Oversimplified Action and Cost Modeling: The partitioning of the action space is frequently too crude, and the assignment of weights to paths relies excessively on idealized states. This approach lacks integration with real-world environmental factors, causing the model formulation to deviate from the actual research problem.
- Neglect of the Path-Planning Model: Research commonly lacks in-depth discussion of the path-planning model itself. The primary focus on proposing a new algorithm leads to the neglect of rigorous processing of environmental map data. Algorithms are typically designed for and directly applied to a given map, or permitted to alter map data. This blurs the responsibility boundary between the algorithm and the map, creating the illusion that traditional or improved algorithms can interfere with the map.
- Over-Reliance on Specific Map: A common technical bottleneck is that the algorithms excessively rely on a single, specific map that must conveniently supply all required data in the correct format. Such an approach overlooks practical scenarios where multiple map sources exist and the data they provide may not directly meet the requirement. Consequently, algorithms may fail to accurately interpret environmental information in real-world settings, limiting their extensibility.

This study is precisely established to address these issues. It employs a dense urban environment characterized by both a 3D environmental map and a 3D radio map as the application scenario. A path-planning model is constructed by synthesizing a complex network, which fuses information from the environmental and radio maps into a format required by the algorithm. Unlike existing approaches that use raw maps directly, this model decouples the algorithm’s pathfinding space from the source maps. It does not rely on a single, specific map and avoids corruption of the original map data. Subsequently, the A* algorithm is enhanced. Compared to traditional A*, it incorporates essential pathfinding constraints to generate feasible, optimal paths that fully comply with all specified limitations. Through a comprehensive scheme that combines the path-planning model with the improved algorithm, this study aims to facilitate and lay a foundation for subsequent research on path planning in complex environments.

## 3. Problem Formulation

### 3.1. Radio Map Model

A radio map is a graphical representation that describes the distribution of radio signal strength, quality, or other parameters in a specific geographic area. Unlike conventional environmental maps that depict elevation, it characterizes the radio environment through features such as signal strength or signal-to-interference noise ratio (SINR), providing a basis for ensuring network connectivity for cellular-connected UAVs. There are two main types of radio maps. One approach is based on propagation-loss models, which employ classical propagation-loss models [46,47,48] to compute signal coverage in space, thereby enabling rapid construction of a radio map. However, these propagation models are theoretical approximations and do not fully match real-world environments, leading to significant deviations between the radio map and the actual environment [49]. The other approach relies on crowdsourced measurements [50], where various radio terminals (e.g., mobile phones, UAVs, vehicle-mounted terminals) measure radio data at different geographic locations, and the data are aggregated in the cloud to generate a more accurate radio map. Considering the high cost of exhaustive measurements, such maps do not require measurements at every location; instead, they use supervised learning or similar methods to interpolate radio parameters for unmeasured positions based on limited measured data, thereby completing the radio map without compromising accuracy [51].

To ensure representativeness, this study adopts a dense urban scenario and employs a crowdsourced-measurement-based radio map that uses the signal-to-interference-plus-noise ratio (SINR) to determine radio-outage probability [13]. The dense urban area is a square region of size D×D, containing *N* base stations, each with multiple cells; the set of cells is denoted as M={1,…,m}. Considering that radio signals transmitted by base stations undergo both large-scale fading and small-scale fading, and given that the positions of base stations and buildings are fixed, it can be assumed that at a given location, large-scale fading is constant while small-scale fading is a small random variable. Assuming that UAVs act as terminals to perform radio measurements at each location, the data obtained are shown in Table 1. The SINR at location $q_{t}$ can then be obtained using Formula (1):

(1) $$\mathrm{SIN}R(q_{t},c(q_{t}))=\frac{p(q_{t},c(q_{t}),\tilde{h}(c(q_{t})))}{\sum_{m\in M}^{m\neq c(q_{t})}p(q_{t},m,\tilde{h}(m))}$$

To assess the radio communication status, a communication-outage event is defined as follows: when a UAV is at location $q_{t}$, the SINR of the signal received from the connected cell $c(q_{t})$ is lower than a given threshold. Based on this, the probability of the communication-outage event is formally represented as:

(2) $$P_{out}(q_{t},c(q_{t}))=\mathrm{Pr}\{\mathrm{SIN}R(q_{t},c(q_{t}))<{\mathrm{SIN}R}_{th}\}$$

where Pr{event} denotes the occurring probability of the corresponding event. At the measurement level, a UAV does not perform a single sample; rather, it collects a large number of independent samples over a sufficiently short observation period. According to the law of large numbers, when the number of measurements is sufficiently large, the sample mean of the outage probability converges to the true value. Therefore, the outage probability for each cell can be calculated based on the mathematical expectation, and the connected cell $c(q_{t})$ can be determined.

Suppose that at time *t,* the UAV is at location $q_{t}$ and performs *J* times of measurements for each cell. Since the measurement duration is sufficiently short, it can be assumed that all measurements are taken at the same location $q_{t}$. When the measurement location is identical, due to the time-invariant nature of large-scale fading, the only factor affecting the measurement results is small-scale fading. Denote the small-scale fading experienced during the *j*-th measurement of cell *m* as $\tilde{h}(m,j)$, and the corresponding measured SINR as $\mathrm{SIN}R(q_{t},m,\tilde{h}(m,j))$. Then the expected outage probability for communication between location $q_{t}$ and cell *m* is:

(3) $$\begin{array}{c} {\bar{P}}_{out}(q_{t},m)=\mathrm{Pr}\{\mathrm{SIN}R(q_{t},m)<{\mathrm{SIN}R}_{th}\}\\ \;\;\;\;\;\;\;\;\;\;\;\;\;\;\;\;\;\;\;=\frac{1}{J}\sum_{j=1}^{J}I\{\mathrm{SIN}R(q_{t},m,\tilde{h}(m,j))<{\mathrm{SIN}R}_{th}\} \end{array}$$

where I{condition} is an indicator function that returns 1 when the specified condition is satisfied and 0 otherwise. The UAV access strategy follows an optimality criterion, i.e., it selects the cell with the minimum expected outage probability as the connected cell. Consequently, in the radio map, the outage probability at location $q_{t}$ is expressed as:

(4) $${\hat{P}}_{out}(q_{t})={\bar{P}}_{out}(q_{t},c(q_{t}))=\underset{m\in \{1,\ldots ,M\}}{\min}{\bar{P}}_{out}(q_{t},m)$$

Following this principle, once a certain number of measurement samples are obtained through crowdsourcing, supervised learning and interpolation estimation can be used to predict radio information for the remaining locations, thereby gradually refining the radio map. Through continuous measurement-and-learning cycles, the prediction results can be verified and calibrated. Considering the practical costs of measurement and computation, it is usually impractical to construct a continuous radio map. Instead, a grid-based spatial map is typically built with a granularity appropriate to the specific requirements of the path-planning task.

### 3.2. Problem Definition

To enable a precise characterization of the research problem, it should be cast as a mathematical optimization problem. This paper focuses on a path-planning scenario where cellular-connected UAVs perform general independent flight missions within a dense urban area characterized by a specific radio map. This scenario primarily corresponds to real-world applications such as area inspection and logistics delivery, where multiple UAVs execute their respective tasks independently while ensuring mutual collision avoidance.

Firstly, in terms of cost, the objective for a UAV is defined as identifying an optimal trajectory from an initial position $q_{S}$ to a final destination $q_{F}$ that satisfies the following conditions:

(1) Minimize the total travel time *T*;
(2) Minimize the likelihood of communication interruptions, as reflected by the outage probability ${\hat{P}}_{out}$

Assuming the UAV operates within a bounded D×D area at a range of altitude *H*, and maintains a constant speed *V*, the cost function for a single UAV can be formalized as the following mathematical model:

(5) $$\begin{array}{ccc} P: & \underset{T,\left\{q_{t}\right\}}{\min}T+\int_{0}^{T}{\hat{P}}_{out}(q_{t})dt & \left(a\right)\\ s.t. & q_{S}=q_{0},\;q_{F}=q_{T}, & \left(b\right)\\ & \left\|{\dot{q}}_{t}\right\|=V,\forall t\in \left[0,T\right], & \left(c\right)\\ & q(\cdot )=[x(\cdot ),y(\cdot ),z(\cdot )], & \left(d\right)\\ & x,y\in [0,D],\;z\in [H_{\min},H_{\max}] & \left(e\right) \end{array}$$

In this formulation, (a) represents the objective function that captures the optimal weighting of the path; (b) specifies the starting and destination locations; (c) enforces the velocity constraint; (d) describes the three-dimensional coordinate representation of each point along the path; and (e) defines the permissible operational region.

**Figure 2:**
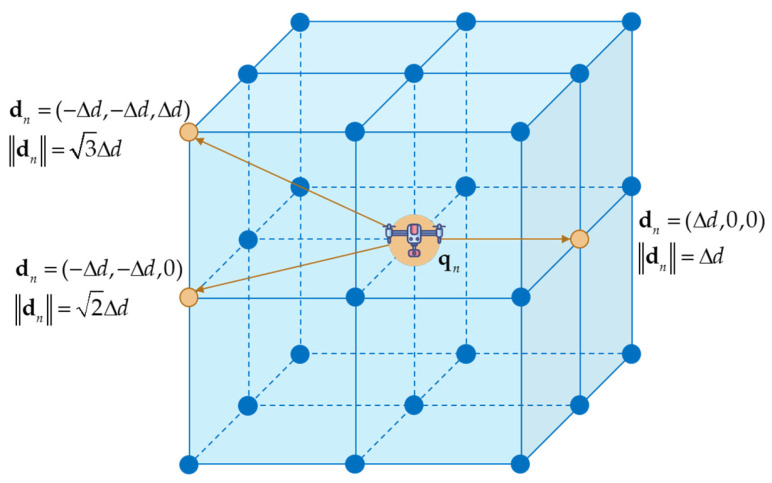
The 26 actions contained in action set A.

Secondly, on the level of the action space, the model presented above applies to a continuous setting. Its non-convex nature, however, poses challenges for direct solution and carries the risk of a dimensionality explosion. Given that the radio map itself possesses a discrete characteristics, and for the quadrotor UAVs, which are typically suitable for dense urban operations, their flight motions are inherently discrete as well, the problem can be converted into a discrete form. Specifically, it is transformed into a navigation problem among nodes of a three-dimensional grid. Using a complex-network model and a discretization step size Δd, the action space is discretized to construct a 3D Cartesian coordinate grid. In this grid, nodes correspond to reachable positions for the UAV, and edges represent the feasible actions the UAV can take in a single step. Defining a single step as a move from the current node to an adjacent grid node, the action set A contains at most 26 directional choices, each with a displacement not exceeding $\sqrt{3}\Delta d$, as illustrated in Figure 2.

.

It is important to note that solving the path-planning problem on a discrete grid exhibits a dependence on the chosen granularity. As Δd decreases, the grid becomes finer, generally improving the accuracy of the planned path. When Δd is sufficiently small, optimizing time becomes equivalent to optimizing distance. Nevertheless, this refinement introduces computational difficulties: if Δd→0, the action space effectively reverts to a continuous one, and the non-convexity causes problem complexity to rise sharply, significantly reducing planning efficiency. Moreover, an excessively fine-grained path may exceed the UAV dynamic constraints and impose impractical demands on the environmental map and radio map. Therefore, selecting Δd is a systematic design choice rather than an arbitrary one. The value should be determined based on factors such as the UAV maneuverability, environmental complexity, and available computational resources, while ensuring the above equivalence condition remains valid. To sum up, the cost model described by Formula (5) can be adapted so that the flight-time component is expressed in terms of distance, with conditions (a) to (c) reformulated as follows:

(6) $$\begin{array}{ll} \underset{N,\left\{q_{n})\right\}}{\min}\sum_{n=0}^{N-1}\left(\left\|q_{n+1}-q_{n}\right\|+{\hat{P}}_{out}(q_{n+1})\right) & \left(a\right)\\ q_{S}=q_{0},\;q_{F}=q_{N}, & \left(b\right)\\ d_{n}=q_{n+1}-q_{n}\in A,\;\left\|d_{n}\right\|\leq \sqrt{3}\Delta d,\;\forall n\in \left[0,N\right] & \left(c\right) \end{array}$$

Thirdly, as for the aspect of multi-UAV constraints, it is further required that the planned paths for multiple UAVs remain conflict-free, meaning UAVs must not collide or approach each other too closely during their missions. A review of existing research reveals diverse approaches to collision avoidance. Examples include UAVs sharing their intent (planned paths, current positions, and goals) via communication, performing collision detection using onboard sensors followed by evasion maneuvers, and employing multi-agent reinforcement learning. While these methods offer dynamic and flexible conflict resolution with high utilization of space and time, they impose stringent requirements on UAV hardware and communication link quality. Safety cannot be guaranteed if hardware or communication fails, and the resulting paths are not necessarily optimal. Alternative methods, such as Conflict-Based Search (CBS), first plan optimal paths independently and subsequently resolve conflicts. These can find globally optimal solutions in cooperative environments and demonstrate favorable theoretical properties. However, in the context of this study, which focuses on a more static path-planning scenario, their advantages are less pronounced. The extended time required for conflict coordination can reduce overall planning efficiency, and the globally optimal solution may not be feasible, posing potential safety risks.

From the perspective of seeking an effective theoretical optimal solution, an absolutely effective and safe method to prevent UAV collisions is to ensure that their paths involve no overlap or intersect. Adhering to this principle, and considering the characteristics of static path planning for multiple UAVs performing relatively independent tasks in this study, a priority-based sequential planning approach emerges as a natural and reasonable choice. Consequently, this paper ensures conflict-free paths among UAVs by marking path occupancy based on a priority scheme. In ultimate summary, the problem model considered in this study is expressed as Formula (7), where (f) represents the path conflict constraint.

(7) $$\begin{array}{ccc} P(u): & \underset{N(u),\left\{q(u,*)\right\}}{\min}\sum_{n=0}^{N(u)-1}\left(\left\|d(u,n)\right\|+{\hat{P}}_{out}(q(u,n+1))\right) & \left(a\right)\\ s.t. & q(u,S)=q(u,0),\;q(u,F)=q(u,N), & \left(b\right)\\ & \begin{array}{l} d(u,n)=q(u,n+1)-q(u,n)\in A,\\ \left\|d(\cdot )\right\|\leq \sqrt{3}\Delta d,\forall n\in \left[0,N\right],\forall u\in U, \end{array} & \left(c\right)\\ & q(\cdot )=[x(\cdot ),y(\cdot ),z(\cdot )], & \left(d\right)\\ & x,y\in [0,D],\;z\in [H_{\min},H_{\max}], & \left(e\right)\\ & \{q(u_{i},*)\}\cap \{q(u_{j},*)\}=\emptyset ,\forall u_{i},u_{j}\in U. & \left(f\right) \end{array}$$

## 4. Methodology

### 4.1. Comprehensive Scheme Architecture

Following problem formulation, a corresponding path-planning algorithm is required to solve it. In general, the performance of graph search algorithms is determined by two key elements: the intrinsic mechanism of the algorithm itself and the definition of the path cost. However, existing research in this area tends to overemphasize the development of path-planning algorithms, with its focus predominantly placed on improving algorithmic mechanisms, while paying insufficient attention to an in-depth discussion of path cost. The design of cost functions is often overly idealized, limiting the practical value of the proposed algorithms for real-world guidance.

Furthermore, existing research often pays insufficient attention to the theoretical underpinnings of the navigation model, and the prevalent practice of tightly coupling the algorithm with the environmental map blurs the boundary of responsibility between the algorithm and the map. In essence, the role of an environmental map model is limited to the static description of space; it is not responsible for formally representing the action space and cost upon which the path-planning algorithm relies. Similarly, the algorithm holds only the responsibility of identifying an optimal path within a specified environment according to the problem model; it should not possess the capability to directly create or modify the underlying environmental description. To fundamentally avoid the series of problems arising from this conflation of responsibility, a dedicated path-planning model is necessary. This model would isolate the algorithm from the environmental map, thereby ensuring the objectivity of the pathfinding environment.

Regarding algorithm applicability, after discretizing the action space into a grid, the form of the path-planning problem aligns well with the search paradigm of the A* algorithm. On the contrary, algorithms designed for continuous action space (such as RRT* from graph theory, which perform random sampling, or DDPG from DRL, where action states are represented as a spatial vector) exhibit poor compatibility with the discrete space. Additionally, the traditional A* algorithm itself has limitations: it focuses solely on finding the minimum-cost path and lacks the capability to accommodate other complex constraints. Although variants of A* exist, such as Hybrid-A* and D* Lite, they only offer advantages within specific, required complex environments instead of being universally applicable, and their optimality and efficiency are inferior to that of A*.

**Figure 3:**
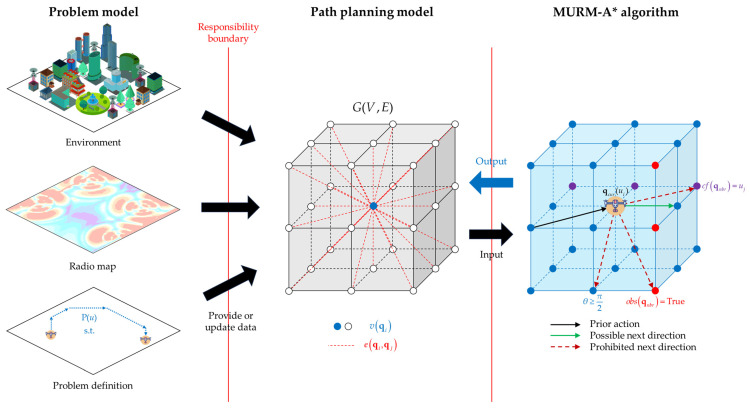
Comprehensive scheme for the problem formulation. A path-planning model is constructed from the problem. MURM-A* is proposed from the path-planning model. The responsibility boundaries are set to ensure that each component only influences itself by receiving data rather than being directly interfered with by other components.

Integrating the above considerations, based on the problem model in Section 3.2, this paper provides a comprehensive multi-UAV path-planning scheme that combines a path-planning model with an improved A* algorithm. The structure of the comprehensive scheme based on the problem model is illustrated in Figure 3.

First, following the problem model and utilizing information from the known environmental map and radio map of the dense urban scenario, a path-planning model is constructed to ensure that the cost setups suffice the requirements of the problem. This approach not only guarantees the authenticity and integrity of the information flow without interfering with the environmental map but also enables timely adaptation to changes in the environmental map information without requiring a complete model reconstruction. Subsequently, based on this path-planning model, the traditional A* algorithm is adopted as the foundational pathfinding component. By integrating it with the multi-UAV path-planning scenario in a radio map environment, an improved A* algorithm named MURM-A* algorithm is proposed.

This architecture clearly delineates the responsibility boundaries between the environmental map, the path-planning model, and the algorithm, ensuring that each component only influences itself by receiving data rather than being directly interfered with by other components. In particular, it prevents the algorithm from overstepping its bounds and performing the functions of the map, thereby protecting the map data from being damaged while the algorithm completes pathfinding.

### 4.2. Path-Planning Model

The construction of a path-planning model aims to reframe the original map scenario into a search space solvable by algorithms, explicitly defining the permissible actions of agents and recording the costs of all potential paths, thereby supplying the necessary pathfinding environment for the algorithm. It is important to clarify that a navigation model is not a universal or broadly applicable standard. In practice, it manifests as a highly tailored solution whose design is strictly dependent on the specific problem definition and the chosen algorithm, resulting in its general applicability only to solving path-planning problems for agents with similar activity patterns in analogous scenarios, and cannot be arbitrarily transferred directly to other algorithmic frameworks. The modeling work in this paper will build a dedicated path-planning model based on a complex network by integrating known environmental information, multi-UAV elements, and the established problem model. In complex-network theory, a graph is typically denoted as G(V,E), where *V* is the set of nodes and *E* is the set of edges, corresponding, respectively, to reachable nodes and available decision actions in the action space. However, the information conveyed by nodes and edges in conventional graphs is often limited. A frequent simplification involves constructing a standardized cost map, wherein various factors and constraints are quantified into cost values to form an integrated cost field, within which search is conducted. Nevertheless, this approach exhibits notable limitations. Primarily, some complex constraints, such as the maneuverability of UAVs, are difficult to incorporate directly into cost maps, often leading to frequent changes in cost values during node expansion and thereby complicating the overall logic. Additionally, determining weight parameters for cost integration is intricate and lacks generalizability, requiring recalibration for different scenarios. More critically, for certain hard constraints, full compliance cannot be guaranteed; in specific situations, constraints may still be forcibly violated, undermining path feasibility.

Considering the inherent directionality of path-planning problems, the requirement to search for cost-optimal paths, and various constraints in multi-UAV scenarios, it is appropriate to employ a graph with directed edges and attachable attributes to construct the path-planning model.

First, a graph with attributes allows the assignment of identifiers and attribute sets to each node and edge. Thus, a node in graph *G* can be represented as $v_{i}(a_{i},l_{i})\in V$ and an edge as $e_{ij}(a_{ij},l_{ij})\in E$. Here, *a* is an identifier, which can be any iterable object used to distinguish or name the node or edge; *l* is an attribute set capable of storing various pieces of information related to the node or edge, and facilitating access by the pathfinding algorithm.

Next, the information associated with each node and edge in the graph is linked to the environmental map, radio map, and problem model to refine the path-planning model. Clearly, for a node $v_{i}$, $a_{i}$ can be set as the corresponding node coordinate $q_{i}=(x_{i},y_{i},z_{i})$. The attributes storable in $l_{i}$ include: (1) the outage probability ${\hat{P}}_{out}(q_{i})$, obtained from the radio map; (2) an obstacle identifier, a Boolean indicator derived from the environmental map showing whether the location falls within an obstacle scope; (3) a conflict identifier, an iterable identifier for judging path conflicts among multiple UAVs, returning the corresponding agent object if the location is occupied by a UAV, otherwise returning a null value. For an edge $e_{ij}$, $a_{ij}$ explicitly specifies the edge direction from position $q_{i}$ to $q_{j}$, denoted as $\left(q_{i},q_{j}\right)$. The primary attributes stored in $l_{ij}$ are: (1) the cost value $w(e_{ij})$, computed synthetically based on node information; (2) a conflict identifier, similar to that for nodes, to determine if a path conflict occurs on the edge. It is important to note that edge directions should be defined according to the UAV’s action set A, and as directed edges, both $e_{ij}$ and $e_{ji}$ can coexist.

After the above organization, the path-planning model used in this paper can be described as follows:

(8) $$\begin{array}{l} G(V,E):\\ \;\;\;\;\forall v(q_{i})\in V:\\ \;\;\;\;\;\;\;\;a=q_{i},\;q_{i}=(x_{i},y_{i},z_{i}),\\ \;\;\;\;\;\;\;\;l=\left\{\begin{array}{l} \mathrm{outage}={\hat{P}}_{out}(q_{i}),\\ \mathrm{obstacle}=obs\left(q_{i}\right),\\ \mathrm{conflict}=cf(q_{i}) \end{array}\right\},\\ \;\;\;\;\forall e(q_{i},q_{j})\in E:\\ \;\;\;\;\;\;\;\;a=(q_{i},q_{j}),\;q_{j}-q_{i}\in A,\\ \;\;\;\;\;\;\;\;l=\left\{\begin{array}{l} \cos t=w(e(q_{i},q_{j})),\\ \mathrm{conflict}=cf((q_{i},q_{j})) \end{array}\right\}. \end{array}$$

Regarding the calculation of the cost $w(e_{ij})$, it must be set strictly according to the cost defined in the problem model, namely:

(9) $$w(e(q_{i},q_{j}))=\lambda_{1}\left\|q_{j}-q_{i}\right\|+\lambda_{2}{\hat{P}}_{out}(q_{j})$$

where $\lambda_{1},\lambda_{2}$ are weight parameters determined by the environment.

### 4.3. MURM-A* Algorithm

Although the traditional A * has theoretical guarantees for the optimality of solutions, it has limitations in complex real-world environments, specifically manifested as: (1) The original design does not incorporate explicit semantic understanding of obstacles or logic for their avoidance; (2) The generated paths may contain sharp turns that violate the kinematic constraints of UAVs; (3) The pathfinding results only consider the planning object itself and cannot handle conflict relationships with other objects in multi-object scenarios. These limitations become unacceptable when facing the inevitable need for building avoidance and UAV collision avoidance when multi-UAVs perform tasks in dense urban low-altitude airspace (approximately 20~60 m).

Therefore, it is necessary to enhance the traditional A* algorithm by embedding essential constraints into the pathfinding process, aiming to output optimal paths that satisfy both communication quality requirements and physical reality with safety regulations. This paper achieves this improvement by deeply integrating the traditional A* algorithm with the radio map environment, thereby proposing a scenario-adaptive enhanced A* algorithm.

It is known that the traditional A* algorithm, as a classic heuristic pathfinding algorithm in static global path planning, primarily accelerates the search process through heuristic estimation, thus significantly improving search efficiency while guaranteeing solution optimality. The heuristic evaluation function for each state *n* is expressed as:

(10) f(n)=g(n)+h(n)

In this context, *g*(*n*) denotes the total incurred cost from the origin to state *n*, while *h*(*n*) represents the projected cost from state *n* to the goal. Within the path-planning model, each state *n* maps to a specific spatial coordinate $q_{n}$. Accordingly, *g*(*n*) is interpreted as the cumulative cost accrued up to that coordinate, and *h*(*n*) is defined as the Euclidean distance from $q_{n}$ to the destination. Then, the heuristic evaluation function within the model framework can thus be concretely expressed as:

(11) $$\begin{array}{c} f(q_{n})=g(q_{n})+h(q_{n}),\\ g(q_{n})=\sum_{i=0}^{n-1}w(e(q_{i},q_{i+1})),\\ h(q_{n})=\left\|q_{n}-q_{F}\right\|. \end{array}$$

With the basic pathfinding logic established, the next step is to consider adding constraints from multi-UAV scenarios and practical factors to the search process to improve the traditional A* algorithm.

(1) Obstacle Avoidance

Considering that the algorithm cannot mark obstacles within the path-planning model beyond the responsibility boundary, a collaborative approach from both the path-planning model and the algorithm mechanism is required. From Formula (8), it is known that the path-planning model has added a Boolean attribute “obstacle” to each node for obstacle identification. This attribute is automatically determined during model construction by querying obstacle data in the environmental map. To simplify the study, only static obstacles like buildings and no-fly zones are considered here. This attribute is then utilized to add node search constraints in the A* algorithm. When the neighbor node position $q_{nbr}$ to be searched falls within an obstacle range, i.e., $obs\left(q_{nbr}\right)=\mathrm{True}$, this node is skipped.

(2) UAV maneuverability Constraints

Considering the common understanding of grid geometry and basic UAV maneuverability, as well as the general need for path smoothness, UAVs have limited turning capability during high-speed maneuvers within the action set A, making it difficult to achieve right angles or sharper turns. Therefore, logical constraints can be imposed on direction changes during the search process, fundamentally eliminating the possibility of excessively sharp turns in the path and ensuring the practical feasibility of the output path for UAVs.

To determine the turning angle of the search direction, three states need to be recorded during the path search: the current state location $q_{cur}$, the previous state location $q_{par}$, and the next neighbor node position $q_{nbr}$ to be searched. The actions for arriving at the current state and moving to the neighbor node are, respectively:

(12) $$\begin{array}{c} \alpha_{cur}=q_{cur}-q_{par},\\ \alpha_{nbr}=q_{nbr}-q_{cur},\\ \alpha_{cur},\alpha_{nbr}\in A. \end{array}$$

Then, the turning angle towards $q_{nbr}$ is calculated as:

(13) $$\theta =\arccos\left(\frac{\alpha_{nbr}\cdot \alpha_{cur}}{\left|\alpha_{nbr}\right|\left|\alpha_{cur}\right|}\right)$$

Specifically, considering that searching from the start point does not perform turning, it is defined that when $q_{cur}=q_{S}$, $\alpha_{cur}=0$, and we have:

(14) $$\theta_{S}=\arccos\left(\frac{\alpha_{nbr}^{2}}{{\left|\alpha_{nbr}\right|}^{2}}\right)=0$$

Accordingly, the pathfinding constraint for UAV maneuverability can be implemented. When searching to $q_{nbr}$, calculate the turning angle *θ*; if $\theta \geq \frac{\pi }{2}$, skip this node.

(3) Multi-UAV Path Conflict Constraints

As mentioned in Section 3.2, for the purpose of seeking an effective theoretical optimal solution among multi-UAVs, conflict-free paths among UAVs are ensured by marking path occupancy relationships. To facilitate managing each UAV, we treat UAV u∈U as an object that encompasses information regarding its identification, starting and ending points, as well as the path:

(15) $$\begin{array}{l} u\in U:\\ \;\;\;\;u.\mathrm{id}=\mathrm{name}\\ \;\;\;\;u.\mathrm{start}=q(u,S)\\ \;\;\;\;u.\mathrm{goal}=q(u,F)\\ \;\;\;\;u.\mathrm{path}=\left\{q(u,*)\right\} \end{array}$$

Considering that path conflicts among UAVs can be categorized into node conflicts and edge conflicts [43], Equation (8) has already set the attribute identifier “conflict” for each node and edge to determine if the corresponding node or edge is occupied by any UAV. The principle is that when constructing the path-planning model, each node $v(q_{i})$ and each edge $e(q_{i},q_{j})$ have their conflict sets defaulted to $cf(q_{i})=\emptyset$, $cf((q_{i},q_{j}))=\emptyset$. Whenever a path is generated for UAV *u* and its task execution begins, the path-planning model marks these nodes and edges as occupied via Equation (16) until the task ends.

(16) $$\begin{array}{c} \forall q(u,n)\in u.\mathrm{path}:cf(q(u,n))=u,\\ \forall q(u,n)\in u.\mathrm{path}-\{q(u,F)\}:cf((q(u,n),q(u,n+1)))=u. \end{array}$$

Correspondingly, the conflict prevention constraints added to the algorithm need to be set for nodes and edges separately. For nodes, when the searched neighbor node $q_{nbr}$ satisfies $cf(q_{nbr})\neq \emptyset$, skip the node. For edges, check other edges intersecting with edge $\left(q_{cur},q_{nbr}\right)$, forming a set $\mathrm{Checklist}(q_{cur},q_{nbr})$. Considering edges intersecting at nodes are already judged by the node condition, only edges intersecting with this edge, where the intersection point is not at a node, need to be included in the set. If $\exists (q_{i},q_{j})\in \mathrm{Checklist}(q_{cur},q_{nbr}):cf((q_{i},q_{j}))\neq \emptyset$, skip the node.

Summarizing all of the above, the process of the MURM-A* algorithm is organized as shown in Algorithm 1.
**Algorithm 1.** MURM-A***Input:** Navigation model *G*, starting point $q_{S}=u.\mathrm{start}$, destination point $q_{F}=u.\mathrm{goal}$.**Begin**Calculate $h(q_{S})$ based on Formula (11), $g(q_{S})\leftarrow 0$, $f(q_{S})\leftarrow h(q_{S})$.OPEN.push([$q_{S}$, ∅, ∅, $g(q_{S})=0$])**While** OPEN≠∅        $\left[q_{cur},q_{par},q_{gpar},g_{pop}(q_{cur})\right]\leftarrow \mathrm{OPEN}.\mathrm{pop}$        **If**
$q_{cur}=q_{F}$ // The destination is reached.            **Return**
$\left\{q(*)\}=\{q_{S},\ldots ,q_{F}.\mathrm{parent},q_{F}\right\}$        **End If**        **If**
$q_{cur}\in \mathrm{CLOSE}$             **If**
$q_{cur}.\mathrm{parent}=\emptyset$ or $g(q_{cur})<g_{pop}(q_{cur})$                  **Continue** // Avoid overriding of parent of starting node or visiting bad path.            **End if**        **Else**             CLOSE.add($q_{cur}$)        **End if**        $q_{cur}.\mathrm{parent}\leftarrow q_{par},q_{par}.\mathrm{parent}\leftarrow q_{gpar}$ // Set the parent node.        **For** each neighbor node $q_{nbr}$ of $q_{cur}$ // Expand nodes.                **If**
$obs\left(q_{nbr}\right)=\mathrm{True}$
                   **Continue** // Skip the node in the obstacle.            **End if**            **If**
$cf(q_{nbr})\neq \emptyset$ or $\exists (q_{i},q_{j})\in \mathrm{Checklist}(q_{cur},q_{nbr}):cf((q_{i},q_{j}))\neq \emptyset$                **Continue** // Skip the node if the path conflicts with other UAVs.            **End if**            Calculate turning angle θ based on Formulas (12)–(14).            **If**
$\theta \geq \frac{\pi }{2}$                **Continue** // Skip the node when the turning angle is excessive.           **End if**
           Calculate $g_{exp}(q_{nbr})$ based on Formula (11).           **If**
$g(q_{nbr})$ has been set and $g_{exp}(q_{nbr})\geq g(q_{nbr})$               **Continue** // Skip the node when a more optimal path to it is already found.           **End if**           Set $g(q_{nbr})\leftarrow g_{exp}(q_{nbr})$, calculate and update $h(q_{nbr})$, $f(q_{nbr})$.           OPEN.push([$q_{nbr}$, $q_{cur}$, $q_{par}$, $g(q_{nbr})$])        **End for****End while****Return** ∅ // Indicating that no path is found.**Output:** Return result (Path set {q(∗)} or ∅)

### 4.4. Systematic Analysis

This section provides further analysis of the aforementioned comprehensive path-planning scheme to facilitate a more precise discussion of its practical applicability.

#### 4.4.1. Extensibility Analysis

Compared to the prevalent approach of directly utilizing environmental maps, the path-planning model within the comprehensive scheme offers enhanced extensibility, primarily demonstrated in the following aspects:

(a) Direct application of algorithms to environmental maps creates a dependency on specific map formats and precision levels. In practice, environmental data often originates from multiple maps whose precision may not align with the granularity required for path planning, and the information they provide is not always directly usable by algorithms. Even minor variations in map format, precision, or semantics can render algorithms incapable of interpreting the data or retrieving necessary information from alternative maps, leading to a significant degradation in performance. In contrast, the proposed path-planning model extracts and transforms relevant information from various maps into a problem-specific representation. This approach eliminates the need for strict adherence to original map data formats and precision, better preserves the integrity of map data, and provides algorithms with a more robust environment for pathfinding.
(b) Real-world environmental maps are not necessarily static; factors within the environment may change, resulting in different environmental states. The path-planning model is grounded in graph theory and does not rely on memorizing specific states. Instead, it depends on an explicit, structured representation of spatial topology and optimization objectives. Consequently, when confronted with changes in environmental conditions, the model itself does not require reconstruction, nor do the core algorithms need modification. Simply updating the model data (e.g., attributes of nodes/edges) enables the algorithms to adapt swiftly to the new state.

#### 4.4.2. Applicability Analysis

Regarding applicability, for the intended positioning of the application, distinctions exist between the proposed path-planning scheme and recent planning systems used in other methodologies, such as radio-map-assisted DRL and graph-based multi-agent framework, as detailed in Table 2.

Subsequently, concerning the specific construction and deployment methodology, the overall workflow of the proposed path-planning scheme is discussed by synthesizing the characteristics of the path-planning model described in Section 4.2 and the MURM-A* algorithm from Section 4.3.

The construction of the path-planning model proceeds through the following steps:

**Input:** Environmental map, Radio map

(1) Construct a graph G(V,E)
(2) According to the problem model, for each position $q_{i}$, add a node $v(q_{i})$ to *V*. Obtain the value ${\hat{P}}_{out}(q_{i})$ from the radio map and store it in the “outage” attribute of $v(q_{i})$. Determine whether $q_{i}$ lies within a building area from the environmental map using a custom-defined rule, and set the “obstacle” attribute based on the result. Initialize $cf(q_{i})=\emptyset$
(3) Following the action set A, add all directed edge $e(q_{i},q_{j})$ to *E*, where the cost value is computed by Formula (9). Initialize $cf((q_{i},q_{j}))=\emptyset$

**Output:** Path-planning model G

It should be added that, given the diverse ways in which real-world maps represent buildings, the rule for identifying buildings should be custom-defined according to the specific environmental map model provided as input, with no universal standard. After the model is constructed, if the environment changes while the problem model remains unchanged, a complete reconstruction is not required; it suffices to inform the path-planning model of the new map data to refresh the relevant attribute.

Based on priority-ordered sequential planning, the scheduling of UAVs can be described as follows:

(1) Prior to mission start, a UAV u∈U submit a request (containing the algorithmic input) and enter the path planning request queue.
(2) Following the queue order, the pathfinding process for each UAV is handled according to the MURM-A* algorithm workflow shown in Table 2.
(3) Once a path is obtained, the UAV *u* commences the mission and notifies the path-planning model to mark the occupancy according to Equation (16). If no path is found, the UAV enters a waiting state and re-enter the request queue.
(4) After UAV *u* reaches its destination, it notifies the path-planning model to release the occupancy.

#### 4.4.3. Sensitivity Analysis

Section 3.2 discussed the sensitivity of the problem definition to the discretization of the action space. Based on the 3D action space, it is recognized that as Δd→0, the action space approximates a continuous space, causing the problem complexity to increase sharply, approximately following an $O(n^{3})$ trend. This sensitivity correspondingly influences various aspects of performance and complexity within the comprehensive scheme, manifesting in the following areas:

(1) Environmental Map and Radio Map: The problem generally requires the granularity of the environmental map and the radio map to be of the form $\frac{\Delta d}{k}(k\in {\mathbb{N}}_{+})$ (the environmental map may be continuous). The sampling scale for constructing the radio map tends to grow approximately as $O(n^{3})$, thereby raising the measurement and computational costs of sampling.
(2) Path Planning Model: The computational effort required for modeling increases roughly as $O(n^{3})$
(3) Pathfinding Algorithm: As the search space expands, the computational growth does not exceed $O(n^{3})$ (the worst case approximates $O(n^{3})$). Furthermore, excess route refinement may lead to overly frequent turning by the UAV.
(4) Amount of UAVs: Owing to the sequential nature of the planning process, when the number of UAVs increases, the overall path-planning complexity in the worst case can grow approximately as $\left|U\right|O(n^{3})$. An excessive number of UAVs may also cause UAVs with lower priority to frequently fail in finding a path or experience a sharp degradation in path quality.

Consequently, Δd cannot be arbitrarily chosen. A more pragmatic approach within the scheme is:

(a) Determine Δd reasonably based on factors such as UAV maneuverability, the scale of the UAV fleet, environmental map precision, and available computational resources, while ensuring the equivalence relationship described in Section 3.2 holds.
(b) Sample and generate the radio map according to the precision defined by Δd. Construct the path-planning model with the granularity of Δd

#### 4.4.4. Limitations Analysis

To ensure the representativeness of this study, several key assumptions were made during the construction of the path-planning model and the algorithm. While these assumptions effectively focus the research on core issues, they inevitably introduce a gap between the modeled scenario and fully realistic conditions. The following explicitly summarizes these assumptions and discusses their limitations regarding practical application guidance.

(a) Static Scenario and Discrete Space: The comprehensive scheme primarily performs planning in a static discrete space based on a radio map and a known environment. This requires the radio environment to remain quasi-static over a sufficiently long period, where the radio map errors are confined to small-scale fading. Furthermore, the current formulation for deployment does not account for dynamic elements such as emergent obstacles, temporary no-fly zones, or time-varying radio conditions, and the A* algorithm still faces considerable challenges in dynamic path planning.
(b) Constant Velocity and Neglected Energy Consumption: The scheme assumes UAVs fly at a constant speed and does not explicitly account for energy consumption. In practical deployment, it may be difficult for a UAV to follow a strictly constant speed due to motion inertia, and the obtained optimal path does not guarantee minimal energy expenditure.
(c) Low Level of Cooperation: The scheme primarily targets priority-based, independent task path-planning scenarios. When an excessive number of UAVs submit simultaneous requests or when tasks require tight cooperation, sequential planning may reduce global solution efficiency, and the quality of the global solution depends heavily on the priority ordering.
(d) Stringent Constraint Mechanism: To guarantee absolute safety and feasibility, the scheme completely avoids obstacles, prevents excessively sharp turns, and resolves multi-UAV conflicts by restricting node expansion during pathfinding. However, this approach presupposes that both the scenario setup and the granularity selection (Δd) are reasonable. This makes the practice deployment should carefully configure the scenario and problem requirements. Otherwise, low-priority UAVs may frequently fail to find a feasible path. Regardless, it is acknowledged that in practical applications, prioritizing UAV safety is often a more common and critical concern than guaranteeing a feasible path always exists from the perspective of the algorithm.

## 5. Evaluation

### 5.1. Evaluation Setup

**Figure 4:**
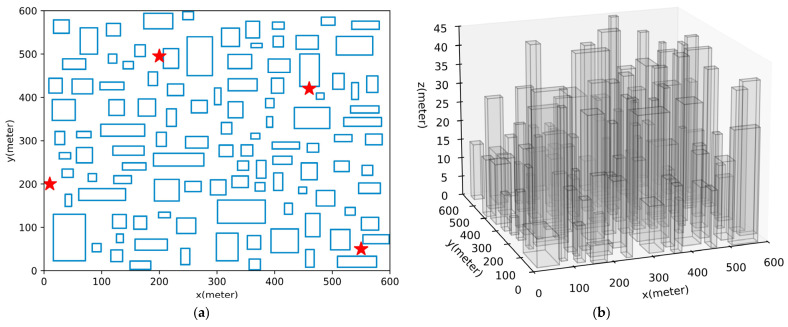

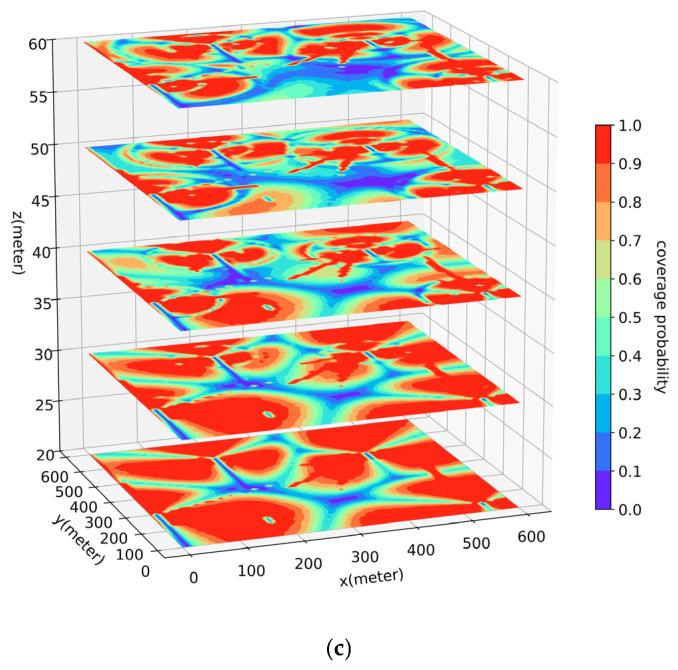
The urban scenario adopted as the evaluation environment. (**a**) 2D preview, where rectangles represent buildings and stars represent base stations; (**b**) 3D preview; (**c**) Corresponding radio map.

This section employs simulation-based comparative experiments to examine the path-planning performance of the proposed MURM-A* algorithm within the constructed path-planning model and to verify the effectiveness of its various constraint-handling capabilities. To ensure the representativeness of the comparative experiments, this paper adopts the method described in the literature [52], which has been validated in real environments, to randomly construct dense urban scenarios and uses their low-altitude airspace as the simulation verification environment. The relevant parameters and descriptions for constructing this urban scenario are provided in Table 3, and an example environmental map, along with the corresponding radio map are shown in Figure 4. The radio map represents the signal coverage probability ${\hat{P}}_{cov}(q_{t})=1-{\hat{P}}_{out}(q_{t})$ at each location based on a granularity of Δd. To ensure consistency of the verification environment, other detailed setups for the scenario and radio map remain consistent with those in [52].

In the experimental design, to reduce complexity, a total of 3 UAVs are set. The start and destination information for each UAV is shown in Table 4. The experiments are divided into two types:

Experiment 1: Performance comparison of algorithms for a single UAV. Under one urban model, conduct 20 experiments for UAV1 and then calculate the average performance of each algorithm (considering minor variations in radio map data due to small-scale fading). The primary goal is to examine the optimality of the algorithm for single-UAV path planning and the modeling efficiency of the path-planning model.

Experiment 2: Comprehensive capability verification of algorithms for multiple UAVs. Randomly generate 20 dense urban models (with base station locations fixed). Under each urban model, conduct 20 experiments involving all UAVs and then calculate the average performance of each algorithm (with randomized priority between UAVs in each experiment). The main purposes are to further examine the overall performance of the algorithm for multi-UAV path planning and to verify its capability in handling path conflicts.

Regarding the selection of comparison objects, to establish performance benchmarks, we compare the proposed algorithm with A* in different conditions. The main comparison objects are the following three:

(1) MURM-A*. The improved A*-based algorithm proposed in this paper, with the principle described in Section 4.3. It will perform pathfinding based on the path-planning model.
(2) PPM-A*. The traditional A* algorithm is incorporated into the path-planning model from Section 4.2 for pathfinding.
(3) EM-A*. The benchmark method is the choice adopted by most of the research. It represents the theoretically optimal solution when traditional A* or MURM-A* performs pathfinding in the environmental map rather than in the path-planning model.

Additionally, extra comparison objects will be added according to each experiment. In Experiment 1, to better demonstrate performance comparisons with methods other than A*, while ensuring fairness and preventing excessive redundancy among comparison objects, after strict screening, the following algorithm, suitable for this experimental scenario, is additionally allowed to join the comparison:

(4) **D3QN**. A state-of-the-art algorithm in the field of DRL [52]. It employs a Markov Decision Process for exploratory training in discrete space, and its training reward mechanism is similar in form to the objective of this research problem. It is designed only for a single UAV.

In Experiment 2, given that after an extensive and meticulous investigation, there are no available directly multi-UAV-oriented external comparable objects that can ensure comparison fairness and be strictly applicable to this scenario, the comparison will instead focus on highlighting the differences between multi-UAV and single-UAV path planning. Therefore, the following comparative object is additionally added:

(5) SURM-A*. Compared to MURM-A*, it only considers the constraints of the single UAV itself.

As for the utilized evaluation metrics setup, the path performance is comprehensively assessed primarily based on UAV flight time and radio-outage time. To evaluate the effectiveness under various constraints, the number of obstacle collisions and sharp turns occurring in the paths is counted. For Experiment 2, the number of conflicts occurring among the paths of all UAVs is also counted. For modeling efficiency, the modeling time is measured, and the pathfinding time is provided for reference.

The simulation experiments are conducted in Python 3.12. Complex-network modeling is implemented using the NetworkX toolkit.

### 5.2. Evaluation Result

**Figure 5:**
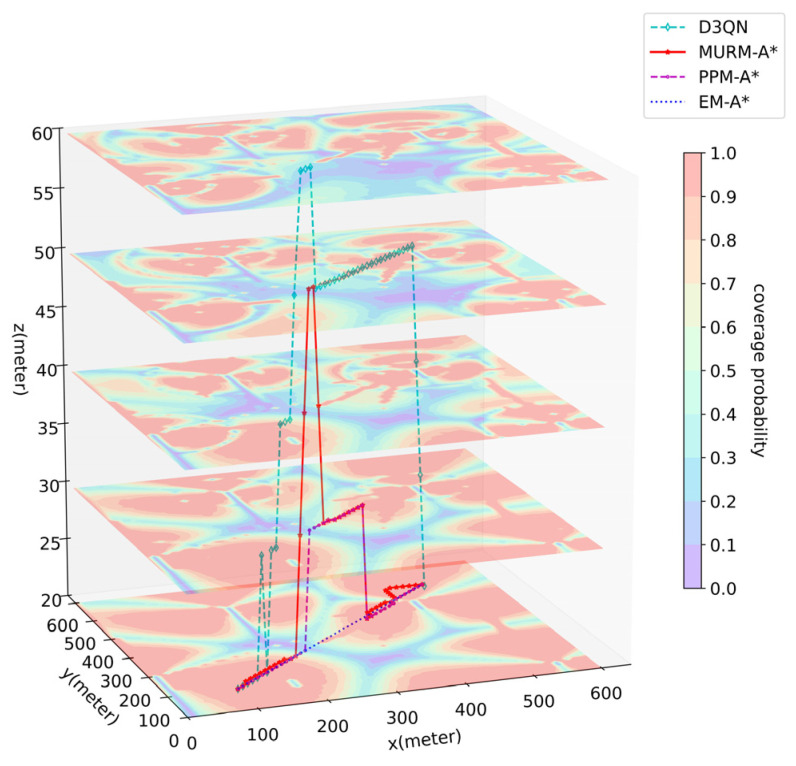
Visualization of a single experiment in Experiment 1.

Experiment 1 compares the performance of algorithms for a single UAV. The path-planning results of each algorithm are shown in Table 5, and the visualized path effects from one of the experiments are selected and presented in Figure 5. Since the maximum building height $h_{obs}=45m>H_{\min}$, there is evidently a large number of densely distributed buildings within the airspace. Moreover, because the low-altitude airspace is close to ground-based stations, the radio coverage is significantly stronger.

The key findings from Experiment 1 are as follows:

- EM-A* disregards environmental and communication constraints, plans the geometrically shortest path (flight time 56.56 s), while exhibiting a high outage duration (average 6.67 s) and a large number of collisions (average 8 times).
- PPM-A* validates the effectiveness of the path-planning model in incorporating communication constraints. Nevertheless, due to the lack of a constraint-handling mechanism, it results in an average of 6.20 obstacle collisions and 0.65 excessive turns.
- D3QN feasibly achieves the shortest flight time (average 59.42 s) but incurs the longest radio outage (average 8.90 s). Moreover, the model training (approximately 60 h) and pathfinding time (average 239.65 s) substantially exceed those of the other methods.
- MURM-A* guarantees 0 obstacle collisions and 0 excessive turns. Compared with D3QN, it increases flight time (around 6%) while reducing outage duration (around 43%). The modeling time remains extremely short (around 2 s).

Regarding the results, EM-A* disregards environmental and communication constraints stemming from its operation without the path-planning model, which prevents it from recognizing obstacles and radio map information. This underscores the necessity of the path-planning model for algorithm performance. After incorporating the path-planning model, the traditional A* algorithm (corresponding to PPM-A*) can jointly consider path length and communication interruption cost. However, it lacks explicit mechanisms for obstacle and maneuverability constraints. The proposed MURM-A* algorithm, building upon traditional A* with introduced constraint-handling mechanisms, completely avoids both collisions and sharp turns, with only a marginal increase in flight time. Although the D3QN algorithm also satisfies the constraints and achieves the shortest flight time among the feasible solutions, its outage time is significantly higher than that of MURM-A*. Moreover, its model training time is substantial, and the pathfinding process itself is also more time-consuming, revealing a notable drawback of DRL methods in terms of extensive training time cost.

In summary of Experiment 1, for the single-UAV scenario, MURM-A* plans feasible, near-optimal paths that achieve zero collisions, zero excessive turns, and significantly reduce radio-outage time compared to D3QN, albeit with a slight increase in flight time. The path-planning model provides essential support for algorithm performance, and both modeling and pathfinding efficiency are substantially higher than the D3QN approach. These findings indicate that the proposed path-planning model and algorithm can effectively balance path length, communication quality, and flight safety, meeting the fundamental requirements for practical cellular-connected UAV operations.

**Figure 6:**
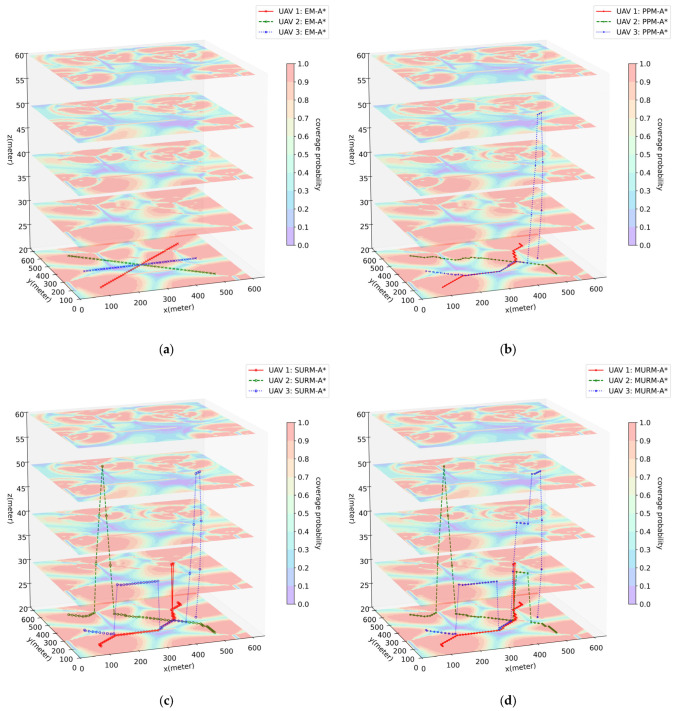
Visualization of a single experiment in Experiment 2. (**a**) EM-A*; (**b**) PPM-A*; (**c**) SURM-A*; (**d**) MURM-A*.

Experiment 2 verifies the comprehensive capabilities of algorithms for multiple UAVs. The path-planning results of each algorithm for all 3 UAVs are shown in Table 6, and the visualized path effects from one of the experiments are selected and presented in Figure 6.

The key findings of Experiment 2 are as follows:

- EM-A* again produces the shortest flight times, yet outage durations are considerably higher (especially for UAV 2 and UAV 3, averaging 15.62 s and 17.64 s, respectively).
- PPM-A* shows improved outage performance, yet frequent obstacle collisions (average 5.49 times) and severe path conflicts (average 10.16 times) persist.
- SURM-A* inherits the communication performance of PPM-A* while achieving 0 collisions and 0 excessive turns. However, it still suffers from a high number of path conflicts (average 7.88 times).
- MURM-A* maintains 0 collisions and 0 excessive turns, reduces path conflicts to 0, and achieves outage durations comparable to those of SURM-A*.
- Model construction times for all algorithms remain on the order of seconds (about 2 s). Due to the additional constraints, MURM-A* exhibits a slight increase in pathfinding time, which is still confined to the millisecond range.

Consistent with Experiment 1, the behavior of EM-A* stems from the planning mechanism, which operates outside the navigation model. Combined with the observation from Figure 6b, PPM-A* tends to guide UAVs to converge on the same Line-of-Sight (LoS) regions, resulting in severe path overlap and a high number of conflicts. Although SURM-A* handles obstacle and turn constraints, it still does not consider multi-UAV conflict avoidance. By integrating a conflict avoidance mechanism, MURM-A* seeks alternative path segments around the optimal ones within LoS regions, ensuring all single-UAV constraints are met while completely avoiding multi-UAV path conflicts. Additionally, from PPM-A* to SURM-A* to MURM-A*, with the stepwise addition of constraints, communication performance and flight efficiency show an incremental trend with minor differences, and pathfinding time increases yet remains at the millisecond level. Summarizing Experiment 2, it corroborates the conclusions from Experiment 1. Furthermore, it reveals that in multi-UAV scenarios, optimizing paths for individual UAVs alone leads to frequent path overlap and conflicts. By introducing a conflict avoidance mechanism, MURM-A* achieves full constraint satisfaction (zero collisions, zero excessive turns, zero conflicts) while maintaining millisecond-level pathfinding time. This validates its effectiveness and safety in multi-UAV scenarios with minimal performance loss.

Synthesizing the above experimental results, the following conclusions can be summarized. Within the same scenario, the path-planning performance achieved using the path-planning model is significantly superior to that obtained by directly using the environmental map. For the path planning of each individual UAV, MURM-A* can incorporate obstacle avoidance and UAV characteristic constraints with only minor deviation from optimality compared to the traditional A* algorithm. Furthermore, compared to the D3QN algorithm, it increases flight time but substantially reduces interruption time and significantly decreases the time required for constructing the path-planning model. For the multi-UAV system as a whole, MURM-A* can comprehensively provide an optimal solution where the paths of all UAVs are conflict-free. It is particularly effective in finding viable alternative solutions to avoid conflicts in situations where multiple UAVs tend to converge towards the same LoS region. These findings indicate that the constructed path-planning model can provide robust computational support for the algorithm. Simultaneously, the MURM-A* algorithm can achieve an effective global joint balance between flight time and radio quality while satisfying all the constraints of the problem. This fully validates that the integrated path-planning scheme, comprising the constructed path-planning model and the proposed MURM-A* algorithm in this paper, is an effective scheme for solving the path-planning problem for multiple connected UAVs in dense urban environments.

## 6. Conclusions

This paper investigates the path-planning problem for multiple cellular-connected UAVs based on 3D radio maps and provides an A*-oriented comprehensive path-planning scheme to obtain optimal solutions. Firstly, an innovative path-planning model was constructed according to the problem scenario and the radio map. Subsequently, under this model, an improved A* path-planning algorithm for multiple UAVs called MURM-A* was proposed. Finally, the proposed algorithm was compared and analyzed against the A* algorithm as well as an external DRL method through comparative simulation experiments conducted in dense urban scenarios. The results show that the MURM-A* algorithm successfully achieves the joint optimization of flight duration and radio connection quality under constraints including static obstacle avoidance, UAV maneuverability limitations, and multi-UAV path conflicts. The effectiveness of the optimal path solutions is significantly superior to that of A* algorithms, which consider only single-UAV constraints, ignore communication quality, or employ traditional search strategies. Moreover, compared to the DRL method, it slightly increases flight time but substantially reduces radio-outage time. As for the constructed path-planning model, it plays a crucial role in enabling the algorithm to identify information within the scenario environment and make accurate pathfinding decisions, and its modeling time is far shorter than the training duration required for DRL models. The experiments validate the effectiveness of the comprehensive path-planning scheme, providing a viable solution for efficiently obtaining optimal path solutions that satisfy multiple constraints.

The path-planning model and algorithmic framework developed in this study possess favorable modularity and extensibility. This foundation facilitates the integration of richer constraint models and more intelligent decision-making mechanisms, paving the way for future research in the following directions:

1. Real-time Replanning in Dynamic Environments: Leveraging the adaptable nature of the path-planning model, future work could focus on incorporating real-time sensor data to dynamically update node/edge attributes, introducing a temporal dimension into occupancy representations, and designing incremental replanning algorithms (e.g., D* Lite) with low computational complexity. This would enable UAVs to respond online to environmental changes and adjust their paths in real time.
2. Integrated Optimization Considering Energy Consumption: A viable approach involves assigning energy cost attributes to all edges based on a kinematic analysis of motion in different directions. However, establishing a precise and universally applicable energy consumption model is challenging due to variations in UAV maneuverability and environmental conditions. Integrating such a model as a key constraint or optimization objective into the existing complex-network-based planning framework remains a complex challenge.
3. Multi-UAV Cooperative Path Planning for Collaborative Tasks: Advancing the path-planning model for cooperative scenarios typically requires redefining nodes from single-agent states to partial or global joint states. Algorithms would then need to evolve beyond mere conflict prevention. They must actively account for the spatiotemporal coupling of UAV trajectories, develop cooperative strategies for task allocation, and optimize communication network topology to achieve true swarm intelligence and maximize collaborative efficiency.

## Figures and Tables

**Table 1:** The obtained measurement data from the UAV.

Element	Description
*t*	Measurement time.
$q_{t}$	Measurement location at time *t*.
$c(q_{t})\in M$	Connected cell, the cell where radio communication is currently conducted with the UAV at location $q_{t}$.
$\tilde{h}(m)$	The small-scale fading in communication between the UAV and the cell *m*. Can be seen as a small random variable.
$p(q_{t},m,\tilde{h}(m))$	The instantaneous signal power measured by the UAV from the cell *m* at position $q_{t}$, containing factors of large-scale fading.

**Table 2:** Comparison between the proposed path-planning scheme and other methodological systems.

Comparison Dimension	Proposed Scheme	Radio-Map-Assisted DRL [13,16]	Graph-Based Multi-Agent Framework [36,43]
Map Input	Environmental map (format-agnostic), radio map	Only a single specific map integrating all information	Only a single specific map matching the format required by the algorithm
Static/Dynamic	Leans towards static	Either static [13] or dynamic [16]	Static planning with dynamic correction
Action Space	Discrete actions	Discrete [13] or continuous [16] actions (depending on DRL framework)	Discrete [43] or continuous [36] actions (depending on adopted algorithm)
Stored Attributes	Recognized and transformed environmental information (obstacles, radio), conflict identifiers, and costs based on problem objectives	Neuron values (e.g., Q-values) in a neural network	Hierarchical storage including cost layer, conflict resolution layer, etc. (no environmental information directly stored)
Planning Paradigm	Priority-based sequential planning	Determined by the DRL framework (e.g., Q-learning)	Centralized parallel planning
Constraint Handling	Restricts node expansion to fully adhere to constraints	Guides agents to obey constraints via a reward function	Plans without constraints initially, followed by conflict detection and explicit constraints resolution
Extensibility	High: No reconstruction needed for environmental changes (only attribute updates required); adaptable to more complex environments by modifying attribute structures	Low: Trained models often exhibit poor generalization, necessitating retraining when encountering environmental changes	Low: Requires reconstruction of the cost layer when environmental changes occur
Optimality and Feasibility	Provides an absolute-feasibility-guaranteed optimal solution for each individual agent. Does not consider global optimality. Overly restrictive constraints may lead to no solution	Typically seeks neuron-value-optimal solutions; performance depends on training quality	Typically guarantees global optimal solutions, but does not ensure feasibility under mandatory constraints, potentially rendering the global optimum invalid
Modeling and Pathfinding Time	Short modeling time, short pathfinding time	Extremely high training cost (long duration, large sample scale)	Short modeling time, while pathfinding time can be long due to conflict resolution mechanisms
Practical Deployment Suitability	Current form suitable for static/slowly changing environments with priority-based, independent task path planning (e.g., urban inspection, logistics delivery). Simple and reliable deployment	Suitable for complex environments requiring online adaptation to unknown radio dynamics. Demands high-fidelity simulation-to-real transfer	Suitable for static/slowly changing environments with cooperative path planning (e.g., warehouse logistics, UAV swarms). Requires strict hardware and radio communication quality

**Table 3:** Relevant parameters for the dense urban scenario.

Parameter	Value
Urban area range *D*	600 m
Maximum height of buildings $\max h_{obs}$	45 m
Aerial area of low altitude	20~60 m
UAV flight speed	10 m/s
Action space granularity	10 m
Environment parameter $\lambda_{1}$	0.1
Environment parameter $\lambda_{2}$	50

**Table 4:** Start and destination information for each UAV u∈U.

u.id	u.start=q(u,S)	u.goal=q(u,F)
UAV1	(100, 100, 20)	(500, 500, 20)
UAV2	(100, 500, 20)	(500, 100, 20)
UAV3	(100, 300, 20)	(500, 300, 20)

**Table 5:** Detailed results of Experiment 1. Average performance of each algorithm after 20 experiments for UAV1.

Method	Average Flight Time (s)	Average Outage Time (s)	Average Obstacle Collisions	Average Excessive Turns	Average Modeling Time (s)	Average Pathfinding Time (s)
EM-A*	56.56	6.67	8.00	0.00	Not applicable	0.01
PPM-A*	57.79	5.10	6.20	0.65	2.44	0.14
D3QN	59.42	8.90	0.00	0.00	222,230.96	239.65
MURM-A*	63.16	5.04	0.00	0.00	2.44	0.22

**Table 6:** Detailed results of Experiment 2. Average performance of each algorithm after conducting 20 experiments in each of 20 randomly generated urban models.

Method	UAV	Average Flight Time (s)	Average Outage Time (s)	Average Obstacle Collisions	Average Excessive Turns	Average Path Conflicts	Average Modeling Time (s)	Average Pathfinding Time (s)
EM-A*	UAV1	56.56	8.88	6.88	0.00	1.00	Not applicable	0.01
	UAV2	56.56	15.62	5.97	0.00			0.01
	UAV3	40.00	17.64	7.10	0.00			0.01
PPM-A*	UAV1	66.61	5.54	6.93	0.63	10.16	2.41	0.08
	UAV2	72.59	6.63	5.05	1.45			0.14
	UAV3	63.53	6.38	4.50	1.95			0.11
SURM-A*	UAV1	68.77	5.65	0.00	0.00	7.88	2.52	0.16
	UAV2	73.55	6.71	0.00	0.00			0.23
	UAV3	64.98	6.76	0.00	0.00			0.23
MURM-A*	UAV1	69.41	5.58	0.00	0.00	0.00	2.58	0.28
	UAV2	75.16	6.82	0.00	0.00			0.38
	UAV3	65.45	6.96	0.00	0.00			0.29

## Data Availability

The original contributions presented in the study are included in the article; further inquiries can be directed to the corresponding author.
